# Neurochemical Properties of the Synapses in the Pathways of Orofacial Nociceptive Reflexes

**DOI:** 10.1371/journal.pone.0034435

**Published:** 2012-03-29

**Authors:** Yu-lin Dong, Wen Wang, Hui Li, Zhi-hong Li, Fu-xing Zhang, Ting Zhang, Ya-cheng Lu, Jin-lian Li, Sheng-xi Wu, Yun-qing Li

**Affiliations:** Department of Anatomy and Histology and Embryology, and K. K. Leung Brain Research Centre, The Fourth Military Medical University, Xi'an, China; University of Salamanca- Institute for Neuroscience of Castille and Leon and Medical School, Spain

## Abstract

The brainstem premotor neurons of the facial nucleus (VII) and hypoglossal (XII) nucleus can integrate orofacial nociceptive input from the caudal spinal trigeminal nucleus (Vc) and coordinate orofacial nociceptive reflex (ONR) responses. However, the synaptoarchitectures of the ONR pathways are still unknown. In the current study, we examined the distribution of GABAergic premotor neurons in the brainstem local ONR pathways, their connections with the Vc projections joining the brainstem ONR pathways and the neurochemical properties of these connections. Retrograde tracer fluoro-gold (FG) was injected into the VII or XII, and anterograde tracer biotinylated dextran amine (BDA) was injected into the Vc. Immunofluorescence histochemical labeling for inhibitory/excitatory neurotransmitters combined with BDA/FG tracing showed that GABAergic premotor neurons were mainly distributed bilaterally in the ponto-medullary reticular formation with an ipsilateral dominance. Some GABAergic premotor neurons made close appositions to the BDA-labeled fibers coming from the Vc, and these appostions were mainly distributed in the parvicellular reticular formation (PCRt), dorsal medullary reticular formation (MdD), and supratrigeminal nucleus (Vsup). We further examined the synaptic relationships between the Vc projecting fibers and premotor neurons in the VII or XII under the confocal laser-scanning microscope and electron microscope, and found that the BDA-labeled axonal terminals that made asymmetric synapses on premotor neurons showed vesicular glutamate transporter 2 (VGluT2) like immunoreactivity. These results indicate that the GABAergic premotor neurons receive excitatory neurotransmission from the Vc and may contribute to modulating the generation of the tonic ONR.

## Introduction

Peripheral orofacial stimuli, such as nociceptive stimulation, can induce the orofacial muscles reflex and corresponding electromyographic (EMG) changes after decerebration [1]–[3]. This indicates the existence of relevant local circuits in the brainstem. These circuits can integrate the peripheral stimuli, transfer them to the brainstem motor neurons and accomplish the reflex activities. Premotor neurons in the brainstem such as the reticular formation and the supratrigeminal nucleus are suggested to be involved in the orofacial nociceptive reflex (ONR) [2], [4]–[5]. We have previously observed the distribution of the premotor neurons of the facial (VII) and hypoglossal (XII) nuclei in the ONR pathway and found that these premotor neurons are mainly distributed bilaterally in the parvicellular reticular formation (PCRt), dorsal and ventral medullary reticular formations (MdD, MdV), supratrigeminal nucleus (Vsup), and parabrachial nucleus (PBN) with an ipsilateral dominance [6].

Gamma-aminobutyric acid (GABA) is an important inhibitory neurotransmitter in the central nervous system (CNS), and most brainstem GABAergic neurons are interneurons. According to previous studies, some premotor neurons of the VII and XII showed GABAergic immunoreactivity [7]–[8]. However, so far, no morphological evidence has been offered supporting that the premotor neurons of the ONR pathways use GABA as their neurotransmitters.

The caudal spinal trigeminal nucleus (Vc) is involved in relaying nociceptive input from the skin of the orofacial region. Previous studies suggested that Vc was not only the region where the trigeminal primary afferents terminated, but also possibly the structure underlying the local pathways of the ONRs [3], [9]. Our previous morphological study showed that the projections originating from Vc made asymmetrical synapses on the premotor neurons of the VII and XII, which suggested that Vc renders excitatory effect on the ONR pathways [6]. However, until now, there has been little morphological evidence regarding the neurochemical properties of these Vc axonal terminals.

Thus, the primary purpose of the present study was to examine the neurochemical features of the synapses on the ONR pathway. Retrograde and anterograde tracing methods were combined with immunofluorescence histochemistry for glutamic acid decarboxylase (GAD), a marker for GABAergic structures, and vesicular glutamate transporter 2 (VGluT2), a marker for the glutamatergic axonal terminals. The double-labeled electronic microscopic experiments were used to further examine the morphologic properties of the synapses formed between Vc projecting fibers and the promoter neurons in the VII and XII.

## Materials and Methods

A total of 25 adult male rats (Sprague-Dawley; China Xi'an, People's Republic of China) weighing 250–300 g were housed in a standard laboratory condition (artificial light cycle 12 h on/12 h off) with food and water *ad libitum*. All experimental protocols and animal care practices were approved by the Institutional Animal Care and Use Committees of the Fourth Military Medical University of China prior to the onset of experiments (Permit number: 10001).

All rats were randomly divided into five groups (5 animals/group) (Table 1). In the first and second groups, BDA, an anterograde tracer, was injected into the Vc. FG, a retrograde tracer, was injected into the ipsilateral VII or XII. Colchicine was injected into the lateral ventricle to determine the relationships between the Vc projecting fibers, the premotor neurons of the VII and XII, and the glutamic acid decarboxylase (GAD) like immunoreactive (LI) neurons. In the third and fourth groups, BDA was injected into the Vc combined with FG into the VII or XII at the same side, respectively. These animals were used to evaluate the relationships among the Vc projecting fibers, vesicular glutamate transporter 2 (VGluT2)-LI axonal terminals, and the premotor neurons in the VII or XII. In the fifth group, electron microscopy was used to examine whether the VGluT2-LI positive axonal terminals projecting from the Vc make synapses on the premotor neurons of the VII or XII by a triple-labeled method after the BDA injection into the Vc and HRP into the VII or XII (Table 1).

**Table 1:** Experimental Cases.

	n	Purpose	Tracer injection sites	colchicine injection
Group I	5	FG/GAD/BDA triple labeling	FG injected into the VII and BDA into the ipsilateral Vc (left side: R1–3; right side: R4–5)	√
Group II	5	FG/GAD/BDA triple labeling	FG injected into the XII and BDA into the ipsilateral Vc (left side: R11–13; right side: R14–15)	√
Group III	5	FG/VGluT2/BDA triple labeling	FG injected into the VII and ipsilateral BDA into the Vc (left side: R6–7; right side: R8–10)	N
Group IV	5	FG/VGluT2/BDA triple labeling	FG injected into the XII and BDA into the ipsilateral Vc (left side: R16–18; right side: R19–20)	N
Group V	5	Electron microscopy	30% HRP injected into the right side VII or XII and BDA into the ipsilateral Vc	N

### Tracer injection and immunohistochemistry study

The animals were deeply anesthetized with sodium pentobarbital (40 mg/kg, i.p.) until no limb-withdrawal reflex was elicited by pinching the tail. They were then placed on a stereotaxic frame and the cisternal cavity of the caudal medulla oblongata was surgically exposed. FG (2%) (catalog number: 80014, Biotium, Hayward, CA, U.S.A) dissolved in 0.05 M phosphate-buffered saline (PBS, pH 7.4) was iontophoresed into the VII or XII with 4 µA positive current pulses (7 s on/7 s off) for 20 minutes, and then an approximate volume of 0.2 µl of a 10% solution of BDA (10,000 MW, catalog number: D1956, Molecular probes, Eugene, OR, U.S.A) dissolved in 0.05 M PBS was injected into the Vc by pressure through a glass micropipette (internal tip diameter = 15–25 µm) which was attached to a 1-µl Hamilton microsyringe.

After FG and BDA injection, the rats were allowed to survive for 5 days. Animals from groups I and II were reanesthetized with sodium pentobarbital (40 mg/kg, i.p.) and injected with 7 µl 1% colchicine into the lateral ventricle. After 12–24 hours of survival, they were deeply anesthetized with sodium pentobarbital (100 mg/kg, i.p.) and perfused transcardially with 100 ml of a solution consisting of 0.9% saline in 0.05 M phosphate buffer (PB, pH 7.4), followed by a volume of 500 ml of 0.1 M PB containing 4% paraformaldehyde and 75% (v/v)-saturated picric acid. The brains were removed and placed in 0.1 M PB containing 30% (w/v) sucrose at 4°C for 24 h. The brainstems were serially cut into transverse sections at 40-µm thickness on a freezing microtome. The sections were divided into four series and collected into 0.05 M PBS (pH 7.4). Animals of groups III and IV were perfused 7 days after FG and BDA injection and cut into 40-µm thickness sections.

The first series of each group was used for observation of the injection site of FG and the distribution of the retrogradely labeled premotor neurons under an epifluorescence microscope (BX-60; Olympus, Tokyo, Japan). The second series was processed for visualization of the injection site of the Vc and the distribution of BDA-labeled fibers in the brainstem. Briefly, the sections were incubated in 0.5% Triton X-100 in 0.05 M PBS (pH 7.6) overnight prior to incubation in the fluorescent isothiocyanate (FITC)-labeled avidin D (1∶200, catalog number: A-2001, Vector Laboratories, Burlingame, CA, U.S.A) at room temperature for 2 h. After the incubation, all sections were rinsed in 0.05 M PBS, mounted onto gelatin-coated glass slides, air-dried, cover-slipped with a mixture of 50% (v/v) glycerin and 2.5% (w/v) triethylenediamine (anti-fading agent) in 0.05 M PBS. Then the injection sites and distribution of BDA-labeled fibers were observed.

The third series of group I–IV (animals which had received exact Vc and VII or XII injections) was used to process FG/GAD/BDA (group I and II) or FG/VGluT2/BDA (group III and IV) triple immunofluorescence labeling. Briefly, the sections were incubated first with a mixture of (1) rabbit anti-FG IgG (1∶5000, catalog number: AB153; Chemicon, Temecula, CA, U.S.A), mouse anti-GAD67 IgG (1∶500, catalog number: MAB5406; Millipore, Temecula, CA, U.S.A) or (2) rabbit anti-FG IgG, guinea pig (GP) anti-VGluT2 (0.5 µg/ml, donated from Kanako T) at room temperature for 12 h, and then with a mixture of FITC-labeled donkey anti-rabbit IgG antibody (1∶500, Catalog number: AP189F; Chemicon, Temecula, CA, U.S.A), Cy5-labeled donkey anti-mouse IgG (1∶500, Catalog number: AP192S; Chemicon, Temecula, CA, U.S.A) or Alexa647-labeled donkey anti-GP IgG (1∶500, Catalog number: A21450;Invitrogen, Eugene, Oregon, U.S.A) and Cy3-labeled avidin D (1∶1000, Catalog number: 016-160-084; Jacson Immuno Research, Newmarket Suffolk, England) for 4 hours. The incubation medium used for the primary antibodies was 0.05 M PBS (pH 7.4) containing 2% normal donkey serum (NDS), 0.5% Triton X-100, 0.05% sodium azide (NaN_3_), and 0.25% λ-carrageen (NDS-PBS). The incubation medium for the secondary antibodies was 0.05 M PBS (pH 7.4) containing 0.5% Triton X-100. For controls, some sections were processed as above, omitting the first anti-GAD or VGluT2 IgG, which resulted in no staining for GAD or VGluT2.

The sections in the fourth series of every group were mounted onto gelatin-coated glass slides and then stained with 1% cresyl violet. Large projection drawings of these cresyl-violet-stained sections were then prepared, and the location of FG/GAD double labeled neuronal cell bodies were plotted on the projection drawings with the aid of a camera lucida attachment. Subsequently, the data were reconstructed into projection drawings of sets of serial sections.

### Electron microscopic study

The rats in Group V, which survived seven days after the injection of BDA, were allowed to survive for an additional 48 h after injection of 0.1 µl 30% HRP (Catalog number: P8375-5KU; Sigma, St. Louis, MO, U.S.A) by pressure through a glass micropipette into the VII or XII. These rats were deeply anesthetized with an overdose injection of sodium pentobarbital (100 mg/kg, i.p.), and then perfused transcardially with 100 ml of 0.025 M PBS (pH 7.4), followed by 500 ml of a fixative consisting of 4% paraformaldehyde and 0.1% glutaraldehyde in 0.1 M PB (pH 7.4). The brains were removed and stored in 0.1 M PB (pH 7.4) containing of 4% paraformaldehyde at 4°C for 2–4 h.

Serial sections of the pons and medulla were cut transversely on a vibratome (Microslicer DTM-1000; Dosaka EM, Kyoto, Japan) at 50-µm thickness. The sections were divided into four series and collected. The first series of sections were processed for the histochemical demonstration of HRP by using the tetramethylbenzidine-sodium tungstate (TMB-ST) method and the HRP reaction products were intensified with DAB/Cobalt/H_2_O_2_ solution . The sections were incubated in 20% normal goat serum (Vector) in 50 mM Tris-buffered saline (TBS, pH 7.4) for 1–2 h followed by the incubation in 1∶50 avidin-biotin-peroxidase (ABC) complex (Catalog number: AP182S; Vector) at room temperature for 6 h and then at 4°C for another 6 h. The HRP injection site and the distribution of premotor neurons were observed under a light microscope.

The second series of sections was incubated with diaminobenzidine (DAB)-nickel-ammonium sulfate for 4 h to reveal the BDA injection site and the distribution of the anterogradely labeled axonal terminals.

The third series of sections was selected for the pre-embedding electron microscopic study. Briefly, after histochemical confirmation of HRP reactive product, the sections were washed several times in 0.1 M PB. Sections were cryoprotected in solutions containing 10% or 20% sucrose in 0.1 M PB for 30 min and then in 30% sucrose and 10% glycerol in 20 mM PB for 3 hours. The sections were freeze-thawed with liquid nitrogen and then incubated in a blocking solution containing 20% normal goat serum (NGS) in 50 mM Tris-buffered saline (TBS; pH 7.3) for 1 hour, followed by incubation with GP anti-VGluT2 (0.8 µg/ml) antibody diluted in TBS containing 1% NGS at 4°C for 12 hours. After washing in TBS, the sections were incubated for 8 hours in 1/100-diluted anti-GP antibody conjugated to 1.4 nm gold particles (Nanoprobes; Stony Brook, NY, U.S.A). The sections were then processed as follows: (1) 1% postfixation with glutaraldehyde in 0.1 mol/L PB for 10 min; (2) silver enhancement with an HQ Silver Kit (Nanoprobes, Stony Brook, NY, U.S.A); (3) incubation with an ABC reagent (Vector Labs, Burlingame, CA, U.S.A) diluted at 1∶50 in TBS for 3 hours at room temperature; (4) visualization of BDA-labeled axonal terminals by incubation with DAB tetrahydrochloride and H_2_O_2_. The sections were then treated with 1% OsO4 in 0.1 M PB for 25 min, stained with 1% uranyl acetate, dehydrated, and flat embedded in Durcupan resin. When the primary antibody was omitted, no VGluT2 immunoreactivity was observed.

### Images acquisition and processing

The BDA-labeled fibers, VGluT2-LI axonal terminals, FG-labeled neurons, and GAD-LI neurons were observed with a confocal laser-scanning microscope (CLSM, FV1000, Olympus, Tokyo, Japan) under laser beams of 490, 590, and 640 nm with the appropriate emission filters for FITC (520 nm), Rh (615 nm), and Cy5 (705 nm). Then the digital images were arranged and modified (15–20% contrast enhancement) in tiff image files. The ultrastructures were observed with an electron microscopy (CM100; Philips, Eindhoven, the Netherlands).

## Results

The cases in which injection sites were centered in the VII, XII, or Vc were taken for the further analysis.

### 1. GAD immunoreactivity in premotor neurons that received Vc projections

**Figure 1:**
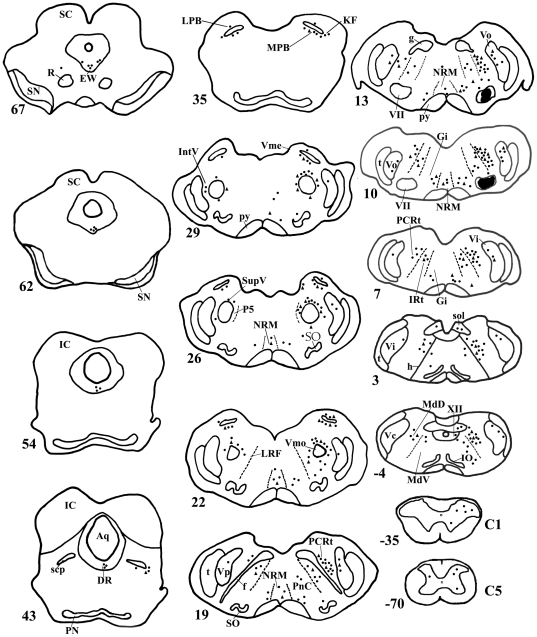
Distribution pattern of the FG/GAD double-labeled neuron after injection of FG into the VII. Projection drawing of transverse section through the brainstem from rat 4, showing the distribution pattern of the FG/GAD double-labeled neuronal cell bodies after the injection of FG into the VII. The injection sites are shown in black. Neuronal cell bodies labeled with FG alone and those labeled with both FG and GAD are represented by filled circles and filled triangles. These are plotted in one-to-one fashion. The numbers indicated the positions of the sections in a series of serial sections of 40 µm thick sections that are arranged rostrocaudally: the number of a section through a level of the obex is indicated by a 0.

**Figure 2:**
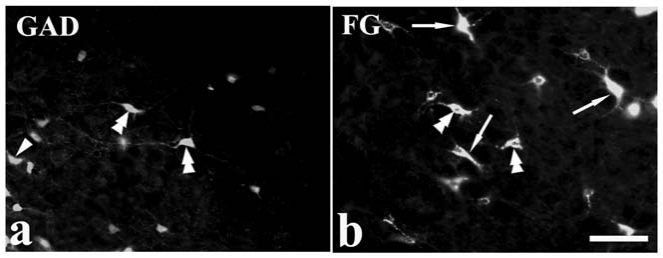
FG/GAD double-labeled neurons. Fluorescent photomicrographs showing (a) GAD-like immunoreactive and (b) FG retrograde-labeled neurons after FG injection into the VII. Double-arrowheads indicate FG/GAD double-labeled neurons, arrowheads and arrows indicate GAD and FG single-labeled neurons, respectively. The photo was taken from the parvicellular reticular formation. Scale bar = 100 µm.

**Figure 3:**
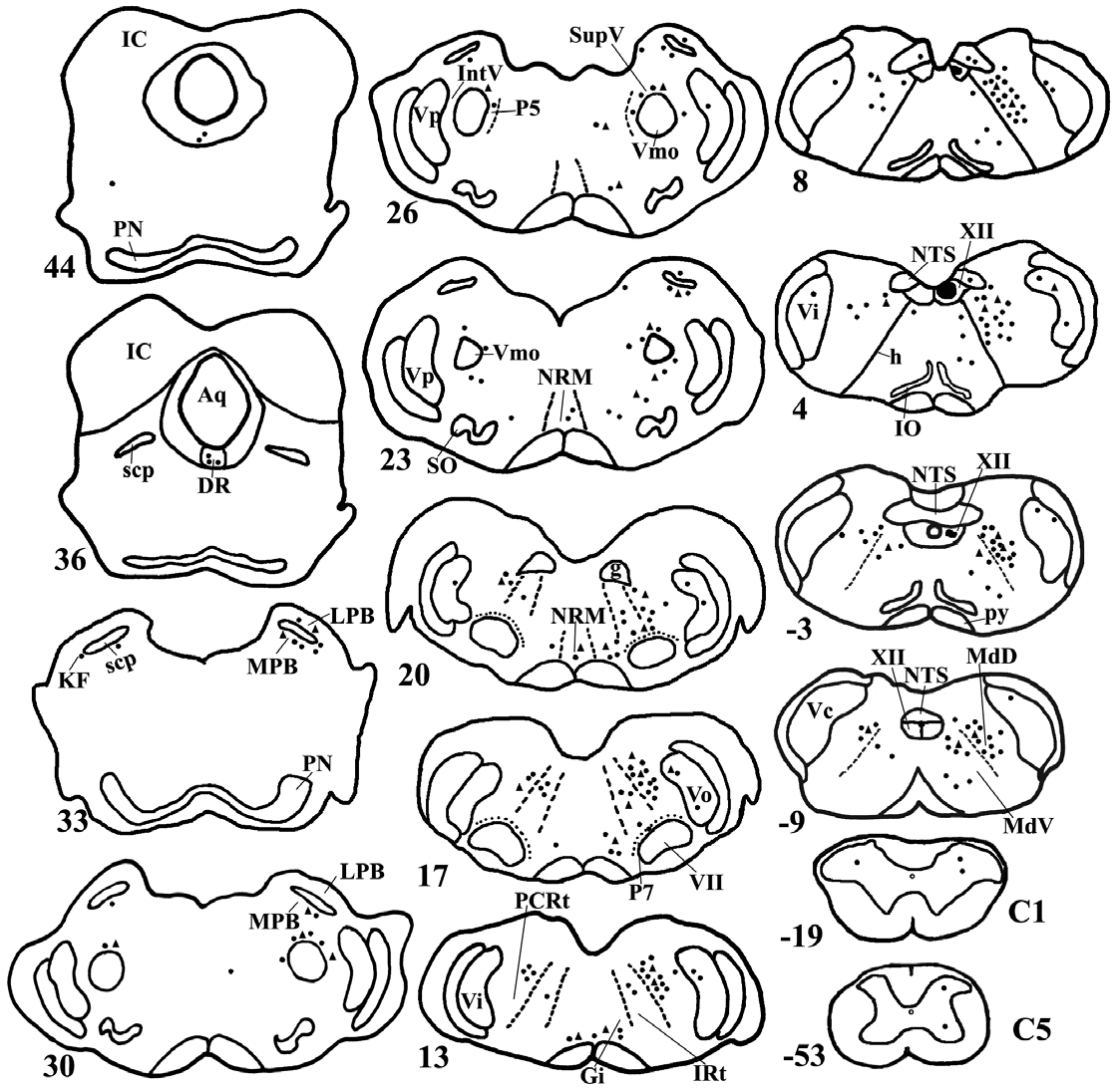
Distribution pattern of FG/GAD double-labeled neurons after injection of FG into the XII. Projection drawing of transverse section through the brainstem of rat 15, showing the distribution patternof the FG/GAD double-labeled neuronal cell bodies with FG injection into the XII. The injection sites are shown in black. The filled circles and filled triangles are plotted in one-to-one fashion, as in Figure 1.

After FG was injected into the VII, the distribution pattern of the FG-labeled premotor neurons was generally consistent with that described in our previous study [6]. Immunofluorescent labeling showed that the FG/GAD double-labeled neurons were distributed bilaterally in the brainstem with an ipsilateral dominance (Fig. 1). The double-labeled neurons were mainly distributed in parvicellular reticular formation (PCRt), dorsal and ventral medullary reticular formation (MdD, MdV), the supratrigeminal nucleus (Vsup), gigantocellular reticular nucleus (Gi), Vo, and Vi (Figs. 1, 2). In addition, some FG/GAD double-labeled neurons were observed in the XII (Fig. 1). After FG was injected into the XII, the distribution of FG/GAD double-labeled neurons was found to be similar to that in the VII injection case (Fig. 3, Table 2).

**Table 2:** Counts of GAD-like immunoreactive neurons that were labeled retrogradely with FG injection into the facial and hypoglossal nucleus.

No. of Rats		PCRt		MdD		MdV		SupV	
		Total FG	FG+GAD(%)	Total FG	FG+GAD(%)	Total FG	FG+GAD(%)	Total FG	FG+GAD(%)
**VII**	R2	127	15(11.8)	89	9(10.1)	71	6(8.5)	56	9(16.1)
	R5	163	21(12.8)	101	9(8.9)	79	8(10.1)	53	10(18.9)
	R7	122	15(12.3)	127	18(14.1)	63	6(9.5)	49	7(14.3)
	R8	139	17(12.2)	97	11(11.3)	87	9(10.3)	49	9(18.8)
	R11	181	29(16.0)	113	15(13.3)	91	13(14.3)	51	6(16.7)
	**Total**	732	97(13.0±1.7)	527	62(11.5±2.2)	391	42(10.5±2.2)	258	41(17.0±1.9)
**XII**	R16	99	13(13.1)	131	18(13.7)	104	9(8.7)	47	9(19.1)
	R17	113	18(15.9)	143	27(18.9)	98	11(11.2)	39	5(12.8)
	R19	78	13(16.7)	169	25(14.8)	139	15(10.8)	36	7(19.4)
	R23	85	11(11.8)	144	15(10.4)	123	17(13.8)	61	10(16.4)
	R25	93	9(9.7)	138	19(13.8)	86	15(17.4)	53	9(17.0)
	**Total**	468	64(13.4±2.9%)	725	104(14.3±3.1%)	550	67(12.3±3.3)	236	40(16.9±2.7)

**Figure 4:**
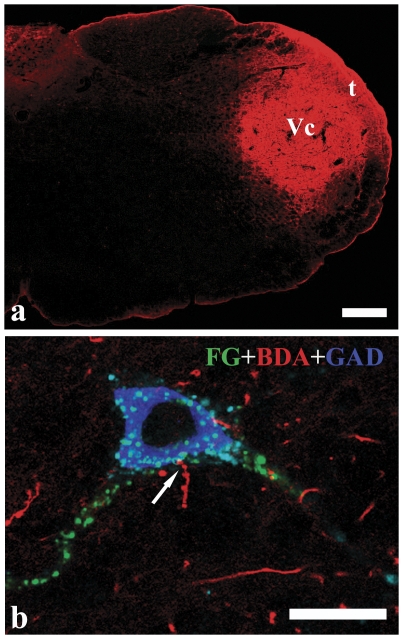
Close appositions between axonal terminals of the Vc and GABAergic premotor neurons of the VII. BDA was injected into the Vc (a). Digital images showing the close appositions (arrows) between axonal terminals (red) of the Vc neurons and FG (green)/GAD (blue) double-labeled neuronal cell body of the premotor neurons in the parvicellular reticular formation(Fig.b). Scale bars = 250 µm in a and 10 µm in b.

After BDA was injected into the Vc, BDA-labeled fibers were observed, bilaterally with an ipsilateral dominance, from the rostral to the ventral area of the brainstem. The labeled fibers and terminals were found to be widely scattered all through the ponto-medullary reticular formation, as in our previous study [6]. Under CLSM, the FG-labeled premotor neurons were shown in green, GAD-LI neurons in blue, and the BDA-labeled Vc projecting terminals in red. A large number of BDA-labeled fibers and terminals surrounded FG/GAD double-labeled premotor neurons and apposed to both the soma and dendritic arborizations of these premotor neurons (Fig. 4). The GABAergic double-labeled premotor neurons that received close appositions from the Vc fibers labeled with BDA were distributed bilaterally in the ponto-medullary reticular formation with a slight dominance on the side ipsilateral to the injection site. The appositions between BDA-labeled fibers and FG/GAD double-labeled premotor neurons were mainly observed in the PCRt, MdD, and the regions surrounding the Vmo or VII, such as Vsup.

### 2. VGluT2 immunoreactivity in Vc axonal terminals that formed asymmetric synapses on premotor neurons of the VII or XII

**Figure 5:**
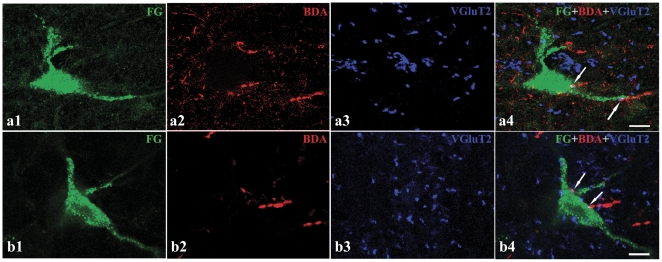
Nociceptive projections showing VGluT2 immunoreactivity from the Vc making close appositions to the premotor neurons of the VII or XII. BDA (red, a2 and b2)/VGluT2 (blue, a3 and b3) double-labeled axonal terminals from the Vc made close appositions (arrows) to the FG retrogradely labeled premotor neurons (green, a1 and b1) after BDA injected into the Vc and FG into the VII (a4) or XII (b4). Both a and b were taken from the ventral medullary reticular formation. Scale bars = 10 µm.

After the injection of FG into the VII or XII and BDA into the Vc, FG-, BDA-, and VGluT2 triple-labeling was performed. VGluT2 immunoreactivity was observed mostly in the varicosities characteristic of axonal terminals in the superficial layer of the Vc, reticular formation , Vsup, and locus ceruleus (LC). Under CLSM, some axonal terminals projecting from the Vc showed VGluT2 immunoreactivity and made close appositions on FG-labeled premotor neurons (Fig. 5).

After injection of BDA into the Vc and HRP into the VII or XII, we examined the relationships among the Vc-projecting axonal terminals, VGluT2-LI buttons, and premotor neurons of the VII and XII using electron microscopy and triple labeling.

**Figure 6:**
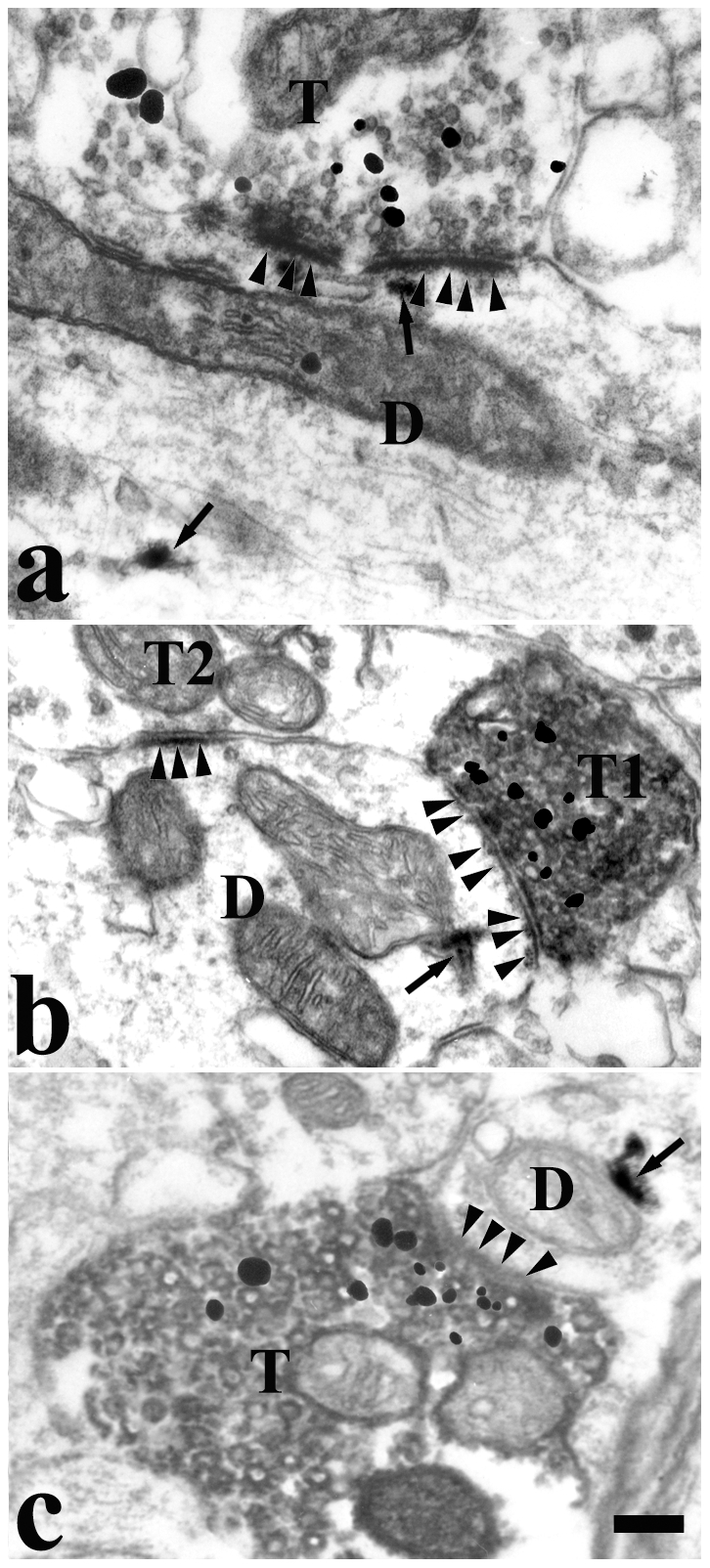
Electron micrograph showing the synapses between the BDA/VGluT2 double-labeled terminals and HRP-labeled dendrites in the brainstem of a rat. HRP was injected into the VII(a and b) or XII (c)of the rat and BDA into the ipsilateral Vc. Note: BDA-labeled terminals were identified by fine granular and homogeneously distributed electron-dense reaction products densely distributed on the membrane of the synaptic vesicles or outer membrane of the mitochondria. Most synaptic vesicles were clear and spherical in their terminal. VGluT2 immunoreactivities were identified by immunogold-silver grains in the presynaptic axonal terminals. HRP-labeled postsynaptic components exhibited highly electron-dense clumps of crystalline or amorphous material (arrows) in the cytoplasm and dendritic profiles. BDA-labeled axonal terminals (T) containing sphere vesicles make asymmetric synapses with the dendrites (D) of HRP-labeled premotor neurons. The arrowheads indicate postsynaptic specializations. (a) was taken from a supratrigeminal nucleus. (b) and (c) were taken from parvicellular reticular formation. Scale bars = 0.3 µm in a; 0.45 in b and c.

HRP-labeled premotor neurons were detected by the presence of highly electron-dense clumps of crystalline material and sometimes by amorphous punctual structures in the cytoplasm of the dendrites and somata. BDA-labeled axonal terminals were ultrastructurally identified by the presence of homogeneously distributed fine, granular, electron-dense cytoplasmic reaction products. These products were located in the cytoplasm and attached to the membranes of the synaptic vesicles and mitochondria. VGluT2 immunoreactivity was determined by the presence of the immunogold-silver grains which were distributed in the cytoplasm of the axonal terminals. Axonal terminals containing more than 4 immunogold-silver grain particles were considered as VGluT2-LI terminals. Under the EM, axonal terminals double-labeled with DAB and immunogold-silver formed asymmetric synaptic contacts with the dendritic profiles and cell bodies retrogradely labeled with HRP (Fig. 6).

## Discussion

In our present study, the neurochemical properties of the synapses on the ONR pathway were examined using tracer tracing combined with immunohistochemistry under both light and electron microscopic levels. These results indicate that some premotor neurons of the VII and XII on the local ONR circuits were GABAergic and received VGluT2-LI axonal projections from the Vc.

### 1. Location of GABA expression in the premotor neurons of the brainstem's intrinsic ONR circuit

Neurons that express GABA are present throughout the CNS and most of them are inhibitory interneurons. Previous morphological experiments have shown that GABAergic axonal terminals are present in the motor nuclei of the brainstem, including the Vmo, VII, and XII [10]. This indicates that the motoneurons received inhibitory projections from other parts of the brain. Li et al. revealed that GABAergic premotor neurons were distributed in the region around the Vmo, medullary reticular formation, trigeminal sensory complex, and raphe magnus using the tracer tracing method combined with immunofluorecent staining [7]–[8]. Electrophysiological studies later showed that stimulation of the regions surrounding the Vmo evoked short-latency excitatory postsynaptic potentials (EPSPs) in masseteric motoneurons and these EPSPs often are either masked by inhibitory postsynaptic potentials (IPSPs) or were followed by long-lasting inhibitory potentials [11]. In addition, the IPSPs were also recorded on the XII neurons after stimulating the lateral reticular formation which has been shown to contain GABAergic and glycinergic neurons that project to the motor nuclei of the brainstem. All these results indicate that the GABAergic inhibitory premotor neurons of the VII and XII are involved in the coordinated activities of the orofacial muscles.

Streppel et al. observed the direct connection between the facial and hypoglossal nucleus at the brainstem level [12]. After injection of a retrograde tracer into the VII, labeled neurons were found in the XII. These neurons appeared small in size, indicating that the GABAergic interneurons in the XII might be involved in the coordination and synchronization of the hypoglossal and facial muscles. Combining FG-retrograde labeling with GAD immunohistochemistry, we offered the direct and further evidence that XII nucleus premotor neurons send out GABAergic inhibitory terminals to the motor neurons in the VII nucleus. These inhibitory connections from XII to VII may play an important role in coordinating orofacial movement in response to orofacial stimuli.

Peripheral nociceptive stimulation can also induce the orofacial muscles reflex. However, so far, no direct morphological evidence has been offered supporting that the GABAergic premotor neurons were distributed on the ONR pathways. Vc is a critical element in the neural pathways underlying ONR [3], [9], [13]. Our group's previous study has shown that, after injection of BDA into the Vc of GAD67-GFP knock-in mice, BDA-labeled axonal boutons form asymmetric synapses on the GABAergic neurons in the Vsup where the premotor neurons locate [5]. In the present study, we further examined the relationship between Vc projection fibers and GABAergic premotor neurons after injection of BDA into the Vc and FG into the VII or XII. The BDA-labeled axonal boutons formed close appositions on GAD-LI neurons retrogradely labeled with FG under the CLSM. It is highly likely that the tonic ONR induced by the orofacial nociceptive stimulation is modulated in this way.

### 2. Formation of asymmetric synapses between the VGluT2-LI axonal terminals projecting from the Vc to the premotor neurons of the VII or XII

Li et al. found that there were many retrogradely labeled neurons in the superficial layer of the Vc after the injection of a retrograde tracer into the Vsup which contains a dense distribution of premotor neurons [5]. They also found that there was no co-localization between the retrogradely labeled neurons and the GABAergic neurons. In our previous study, the Vc axonal boutons formed asymmetric synapses on the premotor neurons of the VII and XII in the brainstem under EM [6]. This indicated that the Vc projecting fibers to premotor neurons release excitatory neurotransmitter, such as glutamate(Glu).

To further examine whether Glu is involved in the transmission of nociceptive information within the ONR pathway, we used VGluT2 to mark glutamatergic axonal terminals. We injected BDA into the Vc and HRP into the VII and XII and observed the relationships among the Vc-projecting fibers, VGluT2-LI axonal terminals, and premotor neurons of the VII and XII. Three kinds of vesicularglutamate transporters, VGluT1, VGluT2 and VGluT3, have been identified and shown to be present selectively in axons belonging to largely non-overlapping populations of glutamatergic neurons throughout the CNS [14]–[16]. The first two of these are specially distributed in the glutamatergic axonal terminals but not in the cell bodies or dendrites. Recent evidence has suggested that VGluT2 is expressed in pain-related pathways and involved in nociceptive transmission on the level of spinal cord and medulla [17]–[19]. We found that BDA-labeled VGluT2-LI axonal terminals from Vc formed asymmetric synapses onto the HRP retrogradely labeled neuronal dendrites within VII and XII. Taking our findings together, our data offered the indirect evidence that Vc sends out excitatory axonal terminals to innervat the GABAergic premotor neurons within VII and XII. Such an excitatory connection makes the ONR intrinsic circuit more complicate and might be more dynamic to coordinate and synchronize the orofacial muscle movements during ONR.

In conclusion, we here provided direct morphological evidence for the neurochemical properties of the synapses within the ONR pathways. Our data suggest that glutamatergic excitatory projections from the Vc can synapse on the GABAergic inhibitory premotor neurons of the VII or XII in the brainstem. These GABAergic premotor neurons are involved in the modulation of nociceptive transmission and contribute to discriminative information processing and inhibition of the occurrence of tonic ONR.
